# Iron Status During Early and Late Pregnancy in a Swedish Multiethnic Population

**DOI:** 10.1155/anem/7110858

**Published:** 2026-10-22

**Authors:** Therese Karlsson, Mathilda Forsby, Anna Hagman, Karin Jerhamre-Sundh, Michael Hoppe, Hanna Augustin

**Affiliations:** ^1^ Department of Internal Medicine and Clinical Nutrition Institute of Medicine Sahlgrenska Academy University of Gothenburg Gothenburg Sweden gu.se; ^2^ Department of Life Sciences Division of Food and Nutrition Science Chalmers University of Technology Gothenburg Sweden chalmers.se; ^3^ Department of Antenatal Care Regional Healthcare Region Västra Götaland Gothenburg Sweden; ^4^ Department of Gastroenterology and Hepatology Clinical Nutrition Unit Sahlgrenska University Hospital Gothenburg Sweden sahlgrenska.se

**Keywords:** iron deficiency, iron status, iron supplementation, pregnancy

## Abstract

**Introduction:**

While ethnic differences in maternal iron deficiency (ID) have been documented in other high‐resource settings, such differences in Sweden remain unknown. This study assessed iron status and the prevalence of ID and anemia in pregnant women from different countries of birth in Sweden.

**Material and Methods:**

This secondary analysis included women enrolled in the randomized controlled Pregnancy and Vitamin D Intervention study (2020–2024;). Blood samples were collected in Trimesters 1 (T1) and 3 (T3), and additional data were obtained from questionnaires and medical records. Women were categorized by region of birth: Europe, Asia, and Africa. Anemia was defined as hemoglobin (Hb) < 110 g/L, and ID was defined using two serum ferritin (SF) thresholds: < 15 and < 30 μg/L.

**Results:**

A total of 128 women (median age 29.8 years) were included in TI, and 89 had blood samples at both T1 and T3. In those with data in both T1 and T3 (*n* = 89), median SF, serum iron, and Hb were significantly lower in T3 compared with T1 (all *p* < 0.001). Between T1 and T3, the prevalence of ID increased from 15% to 39% using SF < 15 μg/L and from 53% to 83% using SF < 30 μg/L (both *p* < 0.001). Anemia prevalence increased from 6% in T1 to 23% in T3 (*p* < 0.001). In T1, women (*n* = 128) from Asia and Africa more often had transferrin receptor concentrations > 4.4 mg/L than women from Europe (*p* = 0.02) but tended only to have a higher prevalence of SF < 15 μg/L (14% and 18% vs. 4%, *p* = 0.06). Self‐reported use of iron supplementation (*n* = 89) increased from early to late pregnancy. According to medical records, 16% of women had been prescribed iron supplements during gestational weeks 6–12 and 85% by Week 32.

**Conclusions:**

ID was common and increased throughout pregnancy in this population in a high‐resource country. No statistically significant differences were observed by region of birth. Despite prescribed iron supplements, nearly one‐quarter were anemic in late pregnancy. This may reflect timing of treatment initiation, adherence, supplement dose, iron absorption, or other unknown contributors, highlighting the need to optimize iron status management during pregnancy.

**Trial Registration:** ClinicalTrials.gov identifier: NCT05329428

**Key Message**
• Iron deficiency was highly prevalent in this multiethnic pregnant population, rising sharply by late pregnancy despite widespread iron supplementation, with similar rates across regions of birth—indicating current preventive strategies may be insufficient to maintain adequate iron status during pregnancy.

## 1. Introduction

Globally, iron deficiency (ID) is the most prevalent micronutrient deficiency, particularly among women of reproductive age and during pregnancy [1]. ID is a contributing factor to fatigue and reduced functional capacity, and it is the most common cause of anemia in pregnancy [1, 2]. This is partly due to increased iron requirements associated with the physiological adaptations of pregnancy [2]. Maternal anemia has been associated with adverse maternal and birth outcomes, including preterm birth, higher rates of cesarean delivery, low birthweight, and small‐for‐gestational age infants [3]. There is less evidence for the association between maternal ID and adverse birth outcomes [4].

There are several biomarkers of iron status, among which serum ferritin (SF) is the most widely used and considered the most reliable single indicator of ID. In the absence of infection or inflammation, ferritin provides a good estimate of iron stores [5]. The primary biomarkers for iron deficiency anemia (IDA) are hemoglobin (Hb) and ferritin. Globally, the prevalence of anemia (of which IDA is the most common) among pregnant women is approximately 35.5%, with regional estimates of 24%–27% in Europe and the United States [6, 7] and the highest rates observed in Southeast Asia (42.1%) and Africa (43.0%) [7, 8]. In Sweden, the overall prevalence of ID during pregnancy has not been well studied and, to our knowledge, remains unclear. In other Nordic countries, the prevalence of ID—defined as SF < 15 μg/L—has been reported to range between 7.9% and 33% in pregnancy [9, 10], with variation attributed to maternal age and ethnic background [9, 11]. Thus, studies from neighboring Nordic countries have reported differences in the prevalence of ID and anemia according to ethnic background, with a higher risk among women who have migrated from low‐ or middle‐income countries or who were born in Asia or Africa [9, 11, 12]. Similar disparities have been observed in the Netherlands, the United States, and Canada [13–15]. However, despite increasing ethnic diversity in many high‐resource countries, including Sweden, data on ethnic and regional differences in maternal iron status remain limited. Whether the disparities observed in other Nordic countries are also present in Sweden is currently unknown.

The World Health Organization recommends routine iron supplementation during pregnancy, with a daily dose of 30–60 mg of elemental iron [16]. Although routine prenatal iron supplementation reduces ID and IDA, its impact on other health outcomes remains less clear [17]. In Sweden, screening for ID is part of standard antenatal care, and iron supplementation at 100 mg/day is recommended when SF is < 30 μg/L in the first trimester [18].

To date, data on the prevalence of ID and anemia among pregnant women of different ethnic backgrounds living in Sweden are limited. Therefore, the aim of the present study was to assess iron status and the prevalence of ID in a multiethnic population of pregnant women in Sweden.

## 2. Materials and Methods

### 2.1. Study Population and Design

The current work was a secondary analysis of longitudinal data collected in the Pregnancy and Vitamin D Intervention (PREDIN) Study. PREDIN is a randomized controlled trial (RCT) designed to evaluate the effects of two different doses of vitamin D supplementation, compared to standard care, on vitamin D status in pregnant women at predicted risk of vitamin D deficiency. This trial is registered at ClinicalTrials.gov. In short, recruitment and data collection were conducted as part of standard antenatal care during the study period. Participants attended two study visits: baseline (Trimester 1 [T1]) and follow‐up (Trimester 3 [T3]). Eligibility criteria included < 15 gestational weeks, maternal age between 18 and 45 years, and being at risk of vitamin D deficiency as determined by a screening questionnaire. Exclusion criteria were multifetal pregnancy, known disorders of vitamin D, calcium, or phosphate metabolism, current vitamin D supplementation (≥ 10 μg/day), and difficulty understanding the study information. A total of 135 women were enrolled at two antenatal clinics in the northeastern region of Gothenburg, an area with low socioeconomic position and a high proportion of immigrant residents. Blood samples and medical records were collected during baseline and follow‐up. Participants also completed questionnaires, which included questions about dietary supplement use. Participants in the PREDIN study were randomized to receive either vitamin D_3_ supplementation at 20 μg/day, 40 μg/day or standard care. Standard care consisted of written and verbal advice on vitamin D supplementation in accordance with current Swedish recommendations for the general adult population. Apart from the vitamin D supplementation advice, antenatal care was similar across all study arms. The study protocol was conducted in accordance with the principles of the Declaration of Helsinki, and the study was approved by the Swedish Ethical Review Authority. All participants gave written informed consent.

Of the 135 enrolled women, two individuals were excluded because they were not eligible for study participation (Figure 1). For the current analysis, five individuals were excluded due to missing blood samples at baseline (*n* = 3), having thalassemia minor (*n* = 1), or being carriers of sickle cell anemia (*n* = 1). A total of 128 individuals with available blood samples from T1 were included. Prior to follow‐up in T3, 39 participants were excluded due to not being at risk for vitamin D deficiency and therefore not included in the PREDIN study and subsequently no samples collected in T2 (*n* = 23), miscarriage (*n* = 7), specialized antenatal care unit (*n* = 1), moving (*n* = 3), drop‐out (*n* = 1), or missing blood samples (*n* = 4). This resulted in a total of 89 participants (hereafter referred to as the “complete set”) who had blood samples collected in both T1 and T3 (Figure 1).

**FIGURE 1:**
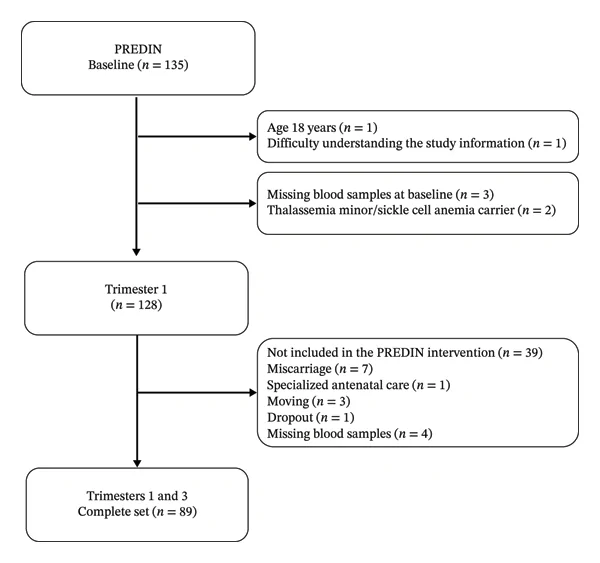
Flowchart of study participants from inclusion in the PREDIN study to the final study group.

### 2.2. Laboratory Analyses and Iron Status Definitions

Nonfasting venous blood samples were collected in T1 and T3. Samples were centrifuged for 10 min at 2200 × g at room temperature within 0.5–2 h after collection and subsequently transported from antenatal care clinics to Sahlgrenska University Hospital. There, study personnel collected samples and processed the samples by aliquoting and storing them at −70°C until further analysis. The samples underwent two freeze–thaw cycles before analysis.

All analyses were performed at the Department of Clinical Chemistry at Sahlgrenska University Hospital, an accredited reference laboratory in compliance with ISO 15189 standards for medical laboratories [19]. All samples were measured using the Abbott Alinity ci analyzer (Abbott Diagnostics, Chicago, USA). Circulating concentrations of Hb, the acute‐phase protein C‐reactive protein (CRP), serum iron, serum soluble transferrin receptor (sTfR), and SF were measured. Total iron‐binding capacity (TIBC) was calculated as transferrin × 25.1, and transferrin saturation (TSAT) was calculated as the ratio of serum iron to TIBC. Blood Hb was measured during standard antenatal care visits using capillary blood samples.

Total body iron (TBI) was calculated from SF and sTfR concentrations according to Cook et al. [20]. Formula used is as follows: −[log10(sTfR × 1000 ÷ SF) − 2.8229 ÷ 0.1207. Positive values of TBI represent storage iron, and negative values indicate a deficient iron supply to peripheral tissues [20].

Anemia in T1 and T3 was defined as Hb < 110 g/L [21]. Given the lack of clear consensus regarding ferritin thresholds for ID, we used two cutoffs for ID analysis: < 15 μg/L according to the World Health Organization [5], and < 30 μg/L, in accordance with United Kingdom, United States, and Swedish guidelines for ID during pregnancy [18, 22, 23]. Ferritin concentrations were not adjusted for inflammation, as there is currently no consensus regarding appropriate inflammation‐adjusted ferritin cutoffs during pregnancy. However, the potential influence of inflammation was explored in separate analyses. ID was also defined as sTfR concentrations > 4.4 mg/L [9, 14] or TBI of < 0 mg/kg [20].

### 2.3. Standard Antenatal Iron Supplementation Recommendations

During the study period (2022–2024), iron supplementation was recommended for women with depleted iron stores. If SF was < 20 μg/L, supplementation was initiated immediately; if ferritin was between 20 and 60 μg/L, supplementation was started around gestational week 20. The recommended dose was 30–50 mg of Fe^2+^ daily. Information on iron supplementation recommendations was retrieved from medical records and categorized as “prescribed iron supplement” or “not prescribed iron supplement.” A woman was classified as “prescribed iron supplement” when the medical records documented that instructions or recommendations for iron supplementation had been provided. Actual use or compliance with iron supplementation could not be ascertained from medical records.

### 2.4. Covariates and Baseline Data

Maternal background information on country of birth and education was collected via a questionnaire during the first trimester. Medical records from antenatal care were obtained after delivery and included details on parity, body weight, height, tobacco use, gestational duration, iron supplementation, and pregnancy complications. At standard antenatal care visits in the first and third trimesters, participants completed a PREDIN study questionnaire that included questions on supplement use. Participants were asked whether they used any supplements and, if so, to provide details on the specific brand, dosage, and frequency. If the information provided was incomplete, study personnel or a midwife contacted the participant to obtain the missing details. The nutrient content of the reported supplements was retrieved from the manufacturers’ or providers’ website. Self‐reported use of iron supplements was defined as *any* or *single* iron supplement. *Any* iron supplement included all supplements containing iron, such as multivitamins, whereas *single* iron supplements were defined as supplements that contained iron only. Data on adherence to self‐reported supplement use were not available.

Body weight (kg) and height (m) were measured during standard antenatal visits, and BMI was calculated as weight divided by height squared (kg/m^2^). Parity was categorized as 1, 2, or ≥ 3. Gestational age was calculated from the first day of the last menstrual period and confirmed by routine ultrasound, as documented in medical records. Ethnicity was not self‐identified. Instead, country of birth was used as a proxy for ethnic background and categorized into Europe, Asia, or Africa, which may not fully capture the ethnic diversity within these groups.

Educational level was categorized as primary school, high school, or university.

### 2.5. Statistics

Continuous variables are presented as median (25th—75th percentiles) and categorical variables as counts (percentages). Spearman’s rank correlation was used to evaluate correlations. A nonparametric test was used due to the relatively small sample size and CRP and ferritin being nonnormally distributed.

Differences in iron status, ID, and anemia between T1 and T3 were assessed using the Wilcoxon signed‐rank test for continuous variables, the McNemar test for dichotomous variables, and the marginal homogeneity test for categorical variables. Differences in iron status, ID, and anemia according to region of birth and CRP levels were analyzed using Fisher’s exact test.

The computer software package SPSS for Windows, Version 29.0 (IBM, NY, USA) was used for statistical analyses. A two‐sided *p* value < 0.05 was considered statistically significant.

## 3. Results

### 3.1. Participant Characteristics

Characteristics of all women are presented in Table 1. For the full cohort (*n* = 128), the median age was 29.8 years (25th–75th percentile: 26.1–34.1), 42% had a university education, and 57% had a BMI above 25 kg/m^2^ in T1. There were no major differences in baseline (T1) characteristics between the full cohort and the complete set (*n* = 89). In the complete set (*n* = 128), region of birth was evenly distributed across Europe, Asia, and Africa. The most common countries of birth were Sweden (Europe), Syria and Iraq (Asia), and Somalia (Africa). Characteristics in T1 by region of birth were similar for age and BMI (Table 1). Women born in Africa had higher parity and a shorter duration of education compared to women born in Europe or Asia.

**TABLE 1:** Trimester 1 characteristics of participants in the PREDIN study, total and by region of birth.

			Region of birth		
	Complete set^a^, *n* = 89	All, *n* = 128	Europe, *n* = 53	Asia, *n* = 37	Africa, *n* = 38
Age (years)	29.9 (26.2–34.2)	29.8 (26.1–34.1)	28.6 (25.7–31.8)	31.2 (28.5–35.3)	30.2 (25.7–34.9)
Gestational week	10.9 (10.2–11.5)	10.7 (10.1–11.4)	10.7 (10.2–11.3)	10.4 (10.0–11.1)	11.0 (10.3–12.0)
BMI (kg/m^2^)	25.5 (22.3–29.1)	25.2 (22.2–29.0)	25.8 (22.2–28.7)	24.5 (22.6–28.4)	26.3 (22.3–31.4)
< 18.5	3 (3.4)	4 (3.6)	1 (2.3)	1 (3.0)	2 (5.6)
18.5–24.9	39 (44.3)	51 (45.5)	20 (46.5)	17 (51.5)	14 (38.9)
≥ 25.0	46 (52.3)	57 (50.9)	22 (51.2)	15 (45.5)	20 (55.6)
Parity					
0	23 (25.8)	42 (35.6)	20 (40.8)	12 (36.4)	10 (27.8)
1	37 (41.6)	44 (37.3)	23 (46.9)	13 (39.4)	8 (22.2)
2	13 (14.6)	16 (12.5)	5 (10.2)	5 (15.2)	6 (16.7)
≥ 3	16 (18.0)	16 (12.5)	1 (2.0)	3 (9.1)	12 (33.3)
Region of birth					
Europe	28 (31.5)	53 (41.4)			
Asia	27 (30.3)	37 (28.9)			
Africa	34 (38.2)	38 (29.7)			
Education					
Primary education	15 (16.9)	18 (14.1)	3 (5.7)	2 (5.4)	13 (34.2)
High school	35 (39.3)	56 (43.8)	27 (50.9)	14 (37.8)	15 (39.5)
University	39 (43.8)	54 (42.2)	23 (43.4)	21 (56.8)	10 (26.3)
Use of tobacco	1 (1.1)	2 (1.6)	2 (4.2)	0 (0)	0 (0)

*Note:* Median (25th–75th percentiles) or proportions (*n* [%]). Missing data complete set (*n* = 89): BMI *n* = 1. Missing data all (*n* = 128): gestational week *n* = 11, BMI *n* = 16, parity *n* = 10, tobacco use *n* = 11.

^a^Participants with data at both Trimesters 1 and 3.

### 3.2. Iron Status

Iron status in T1 and T3 is presented in Table 2. For all (*n* = 128) in T1, median (25th–75th percentile) SF was 34 (22–61) μg/L, serum iron 16 [12–21] μmol/L, and Hb was 128 (122–135) g/L. For the complete set (*n* = 89), median concentrations of SF, serum iron, and Hb were lower in T3 than in T1. ID prevalence defined as SF < 15 μg/L rose from 11% in T1 to 39% in T3 (*p* < 0.001), while SF prevalence of < 30 μg/L increased from 44% to 83% (*p* < 0.001). Using sTfR > 4.4 mg/L or TBI < 0 mg/kg as thresholds for ID yielded lower prevalence of ID than SF < 15 μg/L, but the prevalence increased from T1 to T3. Anemia prevalence rose from 4% in T1 to 23% in T3 (*p* < 0.001). Six women received intravenous iron during pregnancy. Exclusion of these women did not materially alter the results. However, the increase in anemia from T1 to T3 was attenuated, although it remained statistically significant (Supporting Table 1).

**TABLE 2:** Iron status, iron deficiency, and anemia in Trimesters 1 and 3 in the PREDIN study.

	All (*n* = 128)		Complete set^a^ (*n* = 89)				
	Trimester 1	95% CI	Trimester 1	95% CI	Trimester 3	95% CI	*p*
Gestational week	10.7 (10.1–11.4)		10.9 (10.2–11.5)		32.6 (32.3–33.0)		NA

*Biomarker status*							
S‐Ferritin (μg/L)	34 (22–61)	42–56	29 (18–56)	36–51	17 (11–26)	16–30	< 0.001
< 15	14 (10.9)	NA	13 (14.6)	NA	35 (39.3)	NA	< 0.001
15–< 30	42 (32.8)	NA	34 (38.2)	NA	39 (43.8)	NA	
≥ 30	72 (56.3)	NA	42 (47.2)	NA	15 (16.9)	NA	
S‐Iron (μmol/L)	16 (12–21)	16–19	14 (11–20)	14–17	13 (9.0–17)	13–18	0.03
S‐Transferrin (g/L)	2.7 (2.4–3.0)	2.7–2.8	2.8 (2.4–3.1)	2.6–2.9	3.7 (3.3–4.2)	3.6–3.8	< 0.001
S‐Transferrin receptor (mg/L)	2.5 (2.2–2.9)	2.5–2.9	2.7 (2.3–3.0)	2.6–3.0	3.5 (2.9–4.3)	3.5–4.2	< 0.001
S‐Transferrin saturation	0.24 (0.17–0.33)	0.24–0.28	0.20 (0.16–0.29)	0.21–0.26	0.14 (0.09–0.21)	0.14–0.19	< 0.001
S‐Transferrin saturation < 0.20	45 (35.2)	NA	40 (44.9)	NA	65 (73.0)	NA	< 0.001
Total iron‐binding capacity (μmol/L)	68 (60–77)	67–71	70 (61–78)	66–72	92 (82–100)	90–97	< 0.001
Total body iron (mg/kg)	8.3 (5.7–10.4)	7.5–8.6	7.4 (5.2–10.1)	6.9–8.2	4.0 (2.3–6.3)	3.6–4.8	< 0.001
B‐Hemoglobin (g/L)	128 (122–135)	126–130	127 (121–135)	125–129	115 (110–122)	112–117	< 0.001
S‐C‐reactive protein (mg/L)	4.7 (2.3–10.0)	5.8–8.3	5.2 (2.7–11.0)	6.0–8.9	4.4 (2.8–10.0)	5.7–8.5	0.45
≤ 5.0	65 (52.0)	NA	42 (48.3)	NA	48 (53.9)	NA	0.39
> 5.0–10.0	33 (26.4)	NA	23 (26.4)	NA	21 (23.6)	NA	
> 10.0	27 (21.6)	NA	22 (25.3)	NA	20 (22.5)	NA	

*Iron deficiency and anemia*							
S‐Ferritin < 15 (μg/L)	14 (10.9)	NA	13 (14.6)	NA	35 (39.3)	NA	< 0.001
S‐Ferritin < 30 (μg/L)	56 (43.8)	NA	47 (52.8)	NA	74 (83.1)	NA	< 0.001
S‐Transferrin receptor > 4.4 (mg/L)	5 (3.9)		4 (4.5)		21 (23.6)		< 0.001
Total body iron < 0 (mg/kg)	1 (0.8)		1 (1.1)		7 (7.9)		0.03
Anemia	5 (4.2)		5 (5.6)		17 (23.3)		< 0.001

*Note:* Median (25th–75th percentiles) or proportions (*n* [%]). Missing data all (*n* = 128): CRP *n* = 3, Hb *n* = 10. Missing data complete set (*n* = 89): Trimester 1 CRP *n* = 2; Trimester 3 Hb *n* = 16. Statistical tests: Wilcoxon signed‐rank test, McNemar test, or marginal homogeneity test.

^a^Participants with data at Trimesters 1 and 3.

For the complete set (*n* = 89), the prevalence of ID and anemia varied only slightly by region of birth in T3, with no significant differences for most indicators of ID and anemia (Table 3). The prevalence of SF < 15 μg/L was 32% among women born in Europe, 44% in Asia, and 41% in Africa (*p* = 0.66). Similarly, the prevalence of SF < 30 μg/L was common across the regions of birth (82%, 93%, and 77%, respectively; *p* = 0.27). Low TSAT (< 0.20) was also frequent (71%, 70%, and 77%; *p* = 0.87). Only the prevalence of elevated sTfR (> 4.4 mg/L) differed significantly in T3, being more common in women born in Europe (32%) and Africa (29%) compared to Asia (7%) (*p* < 0.05). Anemia prevalence was similar across regions of birth (Europe 22%, Asia 22%, and Africa 26%; *p* = 1.00).

**TABLE 3:** Trimester 1 and 3 iron deficiency and anemia prevalence in the PREDIN study by region of birth.

	Trimester 1 (*n* = 128)			
	Europe, *n* = 53	Asia, *n* = 37	Africa, *n* = 38	*p*
Iron deficiency *n* (%)				
S‐Ferritin < 15 (μg/L)	2 (3.8)	5 (13.5)	7 (18.4)	0.05
S‐Ferritin < 30 (μg/L)	18 (34.0)	19 (51.4)	19 (50.0)	0.17
S‐Transferrin receptor > 4.4 (mg/L)	0 (0)	1 (2.7)	4 (10.5)	0.02
Total body iron < 0 (mg/kg)	0 (0)	0 (0)	1 (2.6)	0.59
S‐CRP (mg/L)				
≤ 5.0	27 (50.9)	21 (60.0)	17 (45.9)	0.06
> 5.0–10.0	18 (34.0)	9 (25.7)	6 (16.2)	
> 10.0	8 (15.1)	4 (14.3)	14 (37.8)	
Anemia	2 (4.1)	1 (3.0)	2 (5.6)	1.00

	**Trimester 3 (*n* = 89)**			
	**Europe *n* = 28**	**Asia *n* = 27**	**Africa *n* = 34**	**p**

Iron deficiency *n* (%)				
S‐Ferritin < 15 (μg/L)	9 (32.1)	12 (44.4)	14 (41.2)	0.66
S‐Ferritin < 30 (μg/L)	23 (82.1)	25 (92.6)	26 (76.5)	0.27
S‐Transferrin receptor > 4.4 (mg/L)	9 (32.1)	2 (7.4)	10 (29.4)	< 0.05
Total body iron < 0 (mg/kg)	2 (7.1)	2 (7.4)	3 (8.8)	1.00
S‐CRP (mg/L)				0.33
≤ 5.0	13 (46.4)	19 (70.4)	16 (47.1)	
> 5.0–10.0	7 (25.0)	5 (18.5)	9 (26.5)	
> 10.0	8 (28.6)	3 (11.1)	9 (26.5)	
Anemia	5 (21.7)	5 (21.7)	7 (25.9)	1.00

*Note:* Proportions (*n* [%]). Missing data (*n* = 128): CRP *n* = 3, Hb *n* = 10. Missing data complete set (*n* = 89): Trimester 3 Hb *n* = 16. Statistical test: Fisher’s exact test.

For the complete set (*n* = 89), approximately 50% of the women had CRP concentrations > 5 mg/L, with no difference observed between T1 and T3 (Table 2). For the full cohort (*n* = 128), the prevalence of elevated CRP did not differ significantly across regions of birth in T1 (*p* = 0.06), although 38% of women born in Africa had CRP > 10 mg/L compared with 14% and 15% among those born in Asia and Europe, respectively (Table 3). There was no difference in the prevalence of ID or anemia among the CRP categories (≤ 5 mg/L, < 5–10 and > 10 mg/L) in either T1 (*n* = 125) or T3 (*n* = 89) (Table 4).

**TABLE 4:** Trimester 1 and 3 iron deficiency and anemia prevalence in the PREDIN study by CRP status.

	Trimester 1 (*n* = 125)			
	CRP ≤ 5.0 *n* = 65	CRP > 5.0–10.0 *n* = 33	CRP > 10.0 *n* = 27	*p*
Iron deficiency *n* (%)				
S‐Ferritin < 15 (μg/L)	8 (12.3)	4 (12.1)	1 (3.7)	0.43
S‐Ferritin < 30 (μg/L)	30 (46.2)	11 (33.3)	12 (44.4)	0.48
S‐Transferrin receptor > 4.4 (mg/L)	4 (6.2)	0 (0)	1 (3.7)	0.41
Total body iron < 0 (mg/kg)	1 (1.5)	0 (0)	0 (0)	1.00
Anemia	4 (6.7)	1 (0.9)	0 (0)	0.50

	**Trimester 3 (*n* = 89)**			
	**CRP ≤ 5.0 *n* = 48**	**CRP > 5.0-10.0 *n* = 21**	**CRP > 10.0 *n* = 20**	**p**

Iron deficiency *n* (%)				
S‐Ferritin < 15 (μg/L)	18 (37.5)	10 (47.6)	7 (35.0)	0.68
S‐Ferritin < 30 (μg/L)	41 (85.4)	19 (90.5)	14 (70.0)	0.20
S‐Transferrin receptor > 4.4 (mg/L)	9 (18.8)	5 (23.8)	7 (35.0)	0.33
Total body iron < 0 (mg/kg)	3 (6.3)	1 (4.8)	3 (15.0)	0.50
Anemia	10 (24.4)	3 (4.1)	4 (5.5)	0.87

*Note:* Proportions (*n* [%]). Missing data (*n* = 125): Hb *n* = 12. Missing data complete set (*n* = 89): Trimester 3 Hb *n* = 16. Statistical test: Fisher’s exact test.

### 3.3. Supplementation

Self‐reported iron supplement use and prescribed iron supplementation increased substantially from T1 to T3 (Table 5). The proportion of women reporting any iron supplement use increased from 44% to 58%, with self‐reported use of single iron supplements rising from 1% to 30% between T1 and T3. According to medical records, 16% of women had been prescribed iron during gestational weeks 6–12, while 85% had been prescribed iron supplements by Week 32. By T3, 7% had received intravenous iron during the pregnancy.

**TABLE 5:** Reported supplement use from questionnaires and prescribed iron from medical records in the PREDIN study.

	Complete set^a^ (*n* = 89)	
	Trimester 1	Trimester 3
Questionnaire		
Iron (mg/day)	0.0 (0.0–15.0)	8.5 (0.0–32.4)
Iron supplement (any)	39 (43.8)	50 (57.5)
Iron supplement (single)	1 (1.1)	26 (29.9)
Medical record		
Gestational Weeks 6–12	14 (15.7)	—
Gestational Week 32	—	76 (85.4)
Intravenous iron	—	6 (6.7)

*Note:* Median (25th–75th percentiles) or proportions (*n* [%]). Missing data Trimester 1: medical record *n* = 3. Missing data Trimester 3: medical record *n* = 3, questionnaire data *n* = 2.

^a^Participants with data at both Trimesters 1 and 3.

## 4. Discussion

In this longitudinal study of pregnant women living in a high‐resource country, ID was highly prevalent and increased from early to late pregnancy, irrespective of region of birth. By T3, the majority of women had low iron stores, and nearly one‐quarter met the criteria for anemia. To the best of our knowledge, this is the first study to assess ID in a multiethnic population of pregnant women in Sweden.

In our study, the overall prevalence of anemia in early pregnancy was comparable to reports from other European and Nordic countries [2, 9, 10] as well as the United States [6, 24]. That ID increased from early to late pregnancy is also consistent with previous findings [9, 25]. However, the reported prevalence of ID varies across studies. Using a ferritin cutoff of < 15 μg/L, the prevalence of ID in our study was lower compared to that reported in a recent Norwegian study [9] but higher than that reported in a U.S. study [25]. We did not observe any substantial differences in ID (regardless of cutoff) or anemia by ethnic background here reported as region of birth. This contrasts with findings from studies conducted in Norway and Denmark, which reported a higher prevalence of ID and anemia among women of non‐European origin [9, 11, 12]. Comparisons between studies are challenging, though, due to differences in sampling methods, ferritin cutoffs, and population characteristics. Although we observed a tendency toward a higher prevalence of ID and anemia among women born in Africa, this did not reach statistical significance. The study population in our study may represent a relatively high‐risk group residing in an area characterized by a lower socioeconomic position, which may partly explain differences compared with other populations. The absence of clear ethnic disparities suggests that ID in pregnancy may be a generalized public health concern rather than one confined to specific subgroups, at least in this setting. This may also reflect the relatively uniform access to antenatal care in Sweden. Furthermore, differences in iron status may be driven more by environmental and structural factors than by biological factors. Variations in ID and anemia may, therefore, reflect differences in socioeconomic conditions, diet, healthcare access, and antenatal care utilization rather than inherent ethnic differences [26]. This is supported by the INTERGROWTH‐21st Project, which found similar Hb distributions across diverse populations when pregnancies were uncomplicated and antenatal care was optimal [27]. The findings that the majority have depleted or low iron stores during late pregnancy highlight that iron status is a challenge within antenatal care in Sweden.

Despite more than 85% of women having been prescribed iron supplements by T3, ID and anemia remained common. At the time of data collection, iron supplementation during pregnancy was recommended for women with ferritin concentrations < 20 μg/L. Currently, in the region where our study was conducted, ferritin is routinely measured at enrolment in maternity care in Sweden, and daily iron supplementation is recommended when SF < 30 μg/L and every other day when SF concentrations are between 30 and 150 μg/L [18]. In T1, 16% of women were prescribed iron supplementation, suggesting that for some women, initiation of supplementation early in pregnancy, or even prior to conception, may be necessary to maintain adequate iron stores. In the present study, “prescribed” was defined as documentation in the medical records indicating that instructions or recommendations for iron supplementation had been provided. No data on adherence were available; therefore, we could not determine whether the prescribed iron supplementation was followed. This should be considered when interpreting the findings. Our findings suggest that prescription of, or instructions to use, iron supplementation alone may be insufficient to prevent ID during pregnancy. This may possibly be explained by suboptimal adherence, discontinuation due to gastrointestinal side effects [28, 29], as well as by the substantial increase in iron requirements during pregnancy, which makes it difficult to fully replete iron stores among women who are already iron deficient [30]. From a clinical perspective, these results highlight the need for improved monitoring of iron supplementation from early pregnancy and throughout pregnancy, as well as further research to optimize iron supplementation regimens and strategies to increase adherence.

Approximately half of the women had elevated CRP concentrations (> 5 mg/L) in both early and late pregnancy, consistent with previous studies [14, 25]. Pregnancy is associated with physiological changes in inflammatory markers, and recent studies suggest that pregnancy‐specific reference intervals for CRP may be higher than those applied in nonpregnant adults [31]. Nevertheless, it is important to bear in mind that inflammation can increase SF independently of iron stores, potentially masking ID. However, we did not observe any significant differences in the prevalence of ID or anemia across the CRP categories (≤ 5, < 5–10, and > 10 mg/L), which is generally consistent with previous reports [20].

Strengths of the present study include its longitudinal design and the inclusion of a multiethnic population receiving standardized antenatal care. The availability of both laboratory data and information on prescribed and self‐reported supplement use further strengthens the findings. In addition, the application of multiple cutoff levels for ID and related biomarkers provides a more comprehensive assessment of iron status in this population.

Several limitations should also be acknowledged. The sample size was relatively small, particularly for subgroup analyses by region of birth, which may have limited statistical power to detect modest differences. Furthermore, women in the PREDIN study were selected based on their risk of vitamin D deficiency. Given the reported association between vitamin D deficiency and ID [32], the study population may not be fully representative of the general pregnant population, potentially limiting the generalizability of our findings. The lack of detailed data on dietary iron intake and adherence to iron supplementation limits our ability to draw conclusions about the causes of persistent ID, including the role of suboptimal compliance. Furthermore, all samples underwent two freeze–thaw cycles, which may have affected certain biomarkers. However, all analyses were performed by an accredited laboratory using standardized methods. Previous studies have reported low‐to‐medium effects of freeze–thaw cycles on iron markers, with a low impact on ferritin concentrations [33, 34].

In conclusion, our findings suggest a high prevalence of ID during pregnancy. By T3, the majority of women had ID. Despite prescribed iron supplements, nearly one‐quarter were anemic in late pregnancy. This may reflect factors such as the timing of treatment initiation, adherence, iron supplement doses, iron absorption, or other unmeasured contributors, highlighting the need for improved management of iron status during pregnancy. No statistically significant differences in ID were observed by region of birth, indicating that ID in this setting does not appear to be confined to specific subgroups.

## Author Contributions

Hanna Augustin was the principal investigator and initiated the study. Mathilda Forsby conducted recruitment and data collection. Therese Karlsson and Hanna Augustin were involved in the formulation of research questions. Anna Hagman and Karin Jerhamre‐Sundh enabled enrolment and study visits within standard antenatal care. Therese Karlsson conducted the statistical analysis and wrote the first version of the manuscript. All authors (Therese Karlsson, Mathilda Forsby, Anna Hagman, Karin Jerhamre‐Sundh, Michael Hoppe, and Hanna Augustin) were involved in interpretation of the results and writing of the final manuscript. Therese Karlsson and Hanna Augustin had primary responsibility for the final content.

## Funding

The study was financed by grants from the Swedish state under the agreement between the Swedish Government and the County Councils, the ALF‐agreement (ALFGBG‐1006111 and ALFGBG‐965649).

## Disclosure

All authors have read and approved the final version of the manuscript. Therese Karlsson had full access to all of the data in this study and takes complete responsibility for the integrity of the data and the accuracy of the data analysis.

## Ethics Statement

The PREDIN study was approved by the Swedish Ethical Review Authority (Dnr: 2021‐03871 and 2024‐07158‐02). All study subjects provided written informed consent, and the study was conducted in accordance with the Declaration of Helsinki.

## Conflicts of Interest

The authors declare no conflicts of interest.

## Supporting Information

Additional supporting information can be found online in the Supporting Information section.

## Supporting information


**Supporting Information** Supporting Table 1. Iron status, iron deficiency, and anemia in Trimesters 1 and 3 in the PREDIN study excluding women receiving intravenous iron.

## Data Availability

The data that support the findings of this study are available on request from the corresponding author. The data are not publicly available due to privacy or ethical restrictions. Requests for data should be made to Hanna Augustin, hanna.augustin@gu.se. Access to data requires approval from the Swedish Ethical Review Authority.
