# Supplementary material for: Iron Status During Early and Late Pregnancy in a Swedish Multiethnic Population
Source: Anemia. 2026 Oct 22;2026:7110858. doi: 10.1155/anem/7110858 (PMC13691429; doi:10.1155/anem/7110858)
Supplement: Supplementary file 1 [file ANEM-2026-7110858-s001.pdf]

## Supplementary material

**Supplementary Table 1**. Iron status, iron deficiency and anemia in trimester 1 and 3 in the PREDIN study excluding women receiving intravenous iron.

**Suppl Table 1**. Iron status, iron deficiency and anemia in trimester 1 and 3 in the PREDIN study excluding women receiving intravenous iron.

| | **Complete set[a] (n=83)** | | |
|---|---|---|---|
| | **Trimester 1** | **Trimester 3** | **p** |
| **Gestational week** | 10.9 (10.3-11.6) | 32.6 (32.3-33.0) | NA |
| ***Biomarker status*** | | | |
| **S-Ferritin (µg/L)** | 29 (21-60) | 17 (11-26) | <0.001 |
| **<15** | 10 (12.0) | 31 (37.3) | <0.001 |
| **15-<30** | 32 (38.6) | 38 (45.8) | |
| **≥30** | 41 (49.4) | 14 (16.9) | |
| **S-Iron (µmol/L)** | 15 (11-20) | 13 (9.1-17) | 0.01 |
| **S-Transferrin (g/L)** | 2.8 (2.4-3.1) | 3.7 (3.3-4.1) | <0.001 |
| **S-Transferrin receptor (mg/L)** | 2.6 (2.3-3.0) | 3.4 (2.9-4.2) | <0.001 |
| **S-Transferrin saturation** | 0.21 (0.16-0.30) | 0.14 (0.09-0.21) | <0.001 |
| **S-Transferrin saturation <0.20** | 34 (41.0) | 61 (73.5) | <0.001 |
| **Total iron-binding capacity (µmol/L)** | 69 (59-78) | 92 (82-100) | <0.001 |
| **Total body iron (mg/kg)** | 7.8 (5.5-10.2) | 4.1 (2.6-6.3) | <0.001 |
| **B-Hemoglobin (g/L)** | 127 (122-135) | 115 (111-122) | 0.04 |
| **S-C Reactive Protein (mg/L)** | 5.1 (2.5-10.0) | 4.4 (2.8-9.3) | 0.76 |
| **≤5.0** | 40 (49.4) | 46 (55.4) | 0.47 |
| **>5.0-10.0** | 23 (28.4) | 20 (24.1) | |
| **>10.0** | 18 (22.2) | 17 (20.5) | |
| ***Iron deficiency and anemia*** | | | |
| **S-Ferritin <15 (µg/L)** | 10 (12.0) | 31 (37.3) | <0.001 |
| **S-Ferritin <30 (µg/L)** | 42 (50.6) | 69 (83.1) | <0.001 |
| **S-Transferrin receptor >4.4 (mg/L)** | 1 (1.2) | 16 (19.3) | <0.001 |
| **Total body iron <0 (mg/kg)** | 0 (0) | 4 (4.8) | 0.13 |
| **Anemia** | 4 (4.8) | 12 (14.5) | 0.04 |

Median ($25^{th}$-$75^{th}$ percentiles) or proportions [*n* (%)]. Missing data complete set (n=89): trimester 1 CRP n=2; trimester 3 Hb n=16. Statistical tests: Wilcoxon signed rank test, McNemar test or Marginal homogeneity Test.

[a]Participants with data at trimester 1 and 3.
